# Dropout in Psychotherapy for Personality Disorders: A Systematic Review of Predictors

**DOI:** 10.1002/cpp.70080

**Published:** 2025-05-05

**Authors:** Francesca De Salve, Chiara Rossi, Elena Gioacchini, Irene Messina, Osmano Oasi

**Affiliations:** ^1^ Department of Psychology Catholic University of Sacred Heart Milan Italy; ^2^ Faculty of Social and Communication Sciences Universitas Mercatorum Rome Italy

**Keywords:** dropout, personality disorders, psychotherapy, reflective functioning, therapeutic alliance

## Abstract

**Introduction:**

Dropout in psychotherapy for personality disorders is a major challenge, affecting treatment efficacy and mental health care delivery. Influenced by patient characteristics, therapist factors and treatment dynamics, dropout remains prevalent. This systematic review identifies predictors of psychotherapy dropout in individuals with personality disorders to inform strategies that enhance treatment engagement.

**Method:**

A systematic search in PsycINFO, PubMed and Scopus identified 22 studies from 1976 articles. Inclusion criteria required DSM/ICD‐based personality disorder assessments and dropout predictors in psychotherapy. Non‐English or non–peer‐reviewed studies were excluded. Screening followed PRISMA guidelines using Rayyan, and study quality was assessed with the Newcastle–Ottawa Scale (NOS).

**Results:**

Dropout rates ranged from 10.4% to 58%, depending on treatment modality and patient characteristics. Younger age, comorbid substance use disorders, emotional dysregulation, distress tolerance difficulties, childhood emotional abuse, therapist turnover and low motivation were significant predictors of dropout. Conversely, strong therapeutic alliances, mindfulness‐based skills and engagement in phone coaching were associated with improved retention. Other relevant factors included low reflective functioning, lower education levels and socio‐economic adversity, such as receiving disability benefits. Only one study identified low reflective functioning as a dropout predictor. Systemic factors, including treatment organization and care coordination, also played a crucial role.

**Conclusions:**

Addressing dropout requires early engagement strategies, therapist continuity and treatment flexibility. Enhancing therapeutic alliance and reflective functioning may be particularly effective in reducing dropout. Systemic improvements, such as better care coordination and accessibility, are crucial for sustaining engagement and improving psychotherapy outcomes for individuals with personality disorders.

**Registration:** PROSPERO number: CRD42024509283

**Summary**

- Early identification of dropout risk factors is essential.
- The therapeutic alliance serves as a critical protective factor against dropout.
- Enhancing reflective functioning may facilitate treatment retention.
- Flexible and individualized treatment approaches promote engagement.
- Structural and organizational factors significantly influence dropout rates.

## 1 Introduction

A diagnosis of personality disorder (PD) is a clinically significant factor that can statistically predict therapeutic dropout (Swift and Greenberg 2012). Swift and Greenberg (2012) define dropout as a client's unilateral termination of therapy before achieving adequate symptom reduction or completing the treatment plan. Personality encompasses an individual's unique and irreplaceable psychological and relational characteristics, which develop dynamically from a genetically determined biological substrate, known as temperament, and are moulded by environmental influences, forming character (Swift and Greenberg 2012). Through this interaction, stable patterns emerge in how an individual perceives, relates to, and interprets both self and environment, influencing behaviour consistently across various contexts. When personality traits become rigid and maladaptive, leading to functional impairment or subjective distress and deviating significantly from cultural norms, they denote a disorder (APA 2001).

PDs are psychopathological forms marked by enduring and maladaptive patterns of behaviour, cognition and inner experience. These patterns affect various areas of an individual's life, including work, school, social functioning and significant relationships. These pervasive patterns diverge markedly from those accepted by the individual's culture (APA 2001).

PDs exhibit high clinical complexity, often presenting in comorbidity with other conditions. Research indicates a prevalence of PDs at 45% in clinical populations (Zimmerman et al. 2005) and 15% in non‐clinical populations (Grant et al. 2004). However, these estimates may be conservative due to many individuals with PDs not seeking treatment (Wampers et al. 2018). A substantial body of scientific literature demonstrates that, compared to patients with other psychiatric diagnoses, those with PDs are less likely to engage with mental health services and more likely to withdraw from them, with dropout rates as high as 80% (Wampers et al. 2018). For example, the meta‐analysis by McMurran et al. (2010) highlights a considerable average dropout rate of 37% among these patients, with unfavourable consequences for both therapists' morale and sense of efficacy, as well as patients' well‐being and adjustment, in addition to the significant financial burden on healthcare systems. However, McMurran et al. (2010) argue that patients with PDs do not appear more inclined to prematurely terminate therapy compared to patients in other diagnostic categories, contrary to the findings of Swift and Greenberg's (2012) meta‐analysis.

In recent decades, a substantial body of research has emerged on the variables implicated in the premature termination of psychotherapy among patients diagnosed with PDs. This body of literature has developed from the idea that identifying predictors of therapy dropout can help tailor treatments to promote sustained therapeutic engagement (McMurran et al. 2010).

Among the therapist‐related factors influencing dropout, countertransference—the emotional and cognitive reactions therapists experience in response to their patients—has gained increasing attention in psychotherapy research. PDs, particularly borderline and narcissistic presentations, are associated with strong countertransference reactions due to their interpersonal difficulties, emotional dysregulation and intense relational dynamics (Betan et al. 2005; Colli and Ferri 2015). Negative countertransference reactions, such as feelings of helplessness, frustration or disengagement, have been linked to therapeutic ruptures and premature termination (Tishby and Wiseman 2022). Conversely, therapists' ability to recognize, regulate and work through countertransference can enhance treatment effectiveness and foster a stronger therapeutic alliance (Gait and Halewood 2019).

Cognitive‐behavioural therapy (CBT), psychodynamic psychotherapies and systemic psychotherapy are the most established and empirically supported treatment approaches for PDs (Leichsenring et al. 2022). CBT‐based interventions, such as dialectical behavioural therapy (DBT) and schema therapy, are widely used for borderline and other PDs and emphasize structured, skill‐based treatment, which may impact dropout differently than other modalities (Cristea et al. 2017; Assmann et al. 2024). Psychodynamic psychotherapies, including mentalization‐based therapy (MBT) and transference‐focused therapy (TFP), address personality pathology through longer term relational work (Volkert et al. 2019; Clarkin et al. 2021). Systemic psychotherapy, while less frequently studied in the context of PDs, is relevant given its focus on interpersonal dynamics, which are often central to dropout risk (Cristea et al. 2017).

Ogrodniczuk et al. (2008) suggested that risk factors should not be equated with barriers to treatment or indicators of untreatability; the study of correlates and predictors of premature therapeutic termination is not aimed at pre‐emptively excluding categories of patients with specific characteristics. On the contrary, early identification of patients at risk for therapeutic dropout can be useful for planning personalized therapeutic pathways that are responsive to patients' needs (Rossi et al. 2023). Timely recognition of risk factors for premature therapy termination can prove valuable in improving adherence to therapy and therapeutic outcomes among patients with PDs (Gamache et al. 2018).

However, despite the richness of the clinical literature on factors associated with therapeutic dropout among patients with PDs, there is a noticeable gap in convergent and definitive empirical findings (Gamache et al. 2018; Oasi et al. 2024). First, existing studies often focus on general dropout rates rather than systematically identifying the predictive factors influencing treatment discontinuation across different therapeutic modalities (McMurran et al. 2010; Gamache et al. 2018). Second, much of the available research is limited by small sample sizes, heterogeneous methodologies and inconsistent definitions of dropout, making it difficult to draw definitive conclusions (Wampers et al. 2018). Third, while individual patient characteristics (e.g., symptom severity and comorbidities) have been explored as dropout predictors, therapist‐related and process‐related factors, such as countertransference reactions and therapeutic alliance ruptures, remain underexamined despite their potential impact on treatment retention (Tishby and Wiseman 2022). Fourth, there is a lack of comparative analyses examining whether dropout patterns differ between structured (e.g., CBT) and relationally focused (e.g., psychodynamic and systemic) approaches, which could inform tailored interventions to enhance treatment adherence (Cristea et al. 2017).

These considerations highlight the need for an updated systematic review to identify the factors associated with therapeutic dropout among individuals with PDs. Focusing specifically on CBT, psychodynamic and systemic therapies ensures a targeted and clinically relevant synthesis, as dropout patterns and contributing factors are likely to vary across different therapeutic modalities. Broadening the scope to include a wider range of approaches, such as humanistic or integrative therapies, would introduce greater heterogeneity, potentially limiting the interpretability of findings. Therefore, this review aims to systematically examine and synthesize the key predictors of dropout from psychotherapy in individuals with PDs.

## 2 Method

To ensure a relatively recent comprehensive overview of the literature, the starting year for article publication was set as 2013, as this period aligns with the publication of DSM‐5 and reflects a phase of evolving diagnostic frameworks in PDs research. However, studies applying PD diagnoses based on any edition of the DSM or ICD are eligible for inclusion. The review protocol was developed following the Preferred Reporting Items for Systematic Reviews and Meta‐Analysis (PRISMA) guidelines and was registered on PROSPERO (number: CRD42024509283, last update 17/09/2024).

### 2.1 Search Strategy

From January to March 2024 (last update October 2024), data sources for relevant publications on empirical studies were gathered via computer‐based searches in the following databases: Cochrane Central Register of Controlled Trails, Google Scholar, Medline, Scopus, PubMed and PsycINFO. Each database was searched independently using a specific iteration research string: (“Dropout”) AND (“Personality disorder”) AND (“Therapeutic Alliance”) OR (“Countertransference”) OR (“Emotional Response”). These strings were selected to encompass a broad range of features related to dropout phenomena and predictive variables that could influence them. Citations were retrieved independently for each iterative search and compiled into a complete list, which was then screened for duplicates and imported into Rayyan (Ouzzani et al. 2016) for the title and abstract screening. The tool aims to improve the efficiency and transparency of systematic reviews and thanks to the blind review function, it allows the convoluted researchers deputed to evaluate the articles to minimize selection bias. To reduce bias, a third independent judge was included to consider articles in which the two main judges did not agree. More details are given in section 2.4.

### 2.2 Inclusion Criteria

Inclusion criteria for studies encompass the following:

- Full‐text papers available in English and published between 2013 and 2024.
- Peer‐reviewed research articles.
- Studies involving dropout occurrences among PD patients undergoing cognitive‐behavioural psychotherapy, psychodynamic psychotherapies or systemic psychotherapy.
- Longitudinal and experimental studies examining the relationship between dropout and predictive variables of the phenomena (e.g., therapeutic alliance, countertransference, emotional response, patient interpersonal problems, childhood trauma and comorbidities) in individuals with a PD.
- Dropout will be operationalized through one of the following criteria: (a) number of attended therapy sessions, (b) premature termination (i.e., termination before recovery) or (c) unilateral termination (i.e., termination without therapist collaboration on the decision).
- Studies utilizing DSM or ICD criteria and/or structured personality assessments.

### 2.3 Risk of Bias and Quality Assessment

To ensure accuracy and minimize bias, two reviewers (F.D.S. and E.G.) independently screened all nonduplicate titles and abstracts to identify eligible studies. They then conducted a full‐text review of the selected articles. Discrepancies were initially discussed between the two reviewers and resolved through consensus. If consensus could not be reached, a third reviewer (C.R.) acted as an arbitrator to facilitate resolution. This multistep approach ensured the reliability and consistency of the extracted data.

Moreover, the methodological quality of the studies included was ensured using the Newcastle–Ottawa Scale (NOS) a widely used tool in metaresearch (Catalan et al. 2021; Fiorentino et al. 2024). This instrument evaluates the quality of both randomized and nonrandomized comparative studies by examining critical parameters such as reporting strategies, external validity, internal validity and statistical power. Any discrepancies or uncertainties were resolved by consensus between the two reviewers. The findings from the risk of bias assessment are presented in Table 1.

## 3 Results

From the previously mentioned databases, 2082 studies were retrieved, of which 1976 did not meet the preliminary inclusion criteria. Subsequently, 98 full‐text articles were analysed for specific inclusion criteria. Out of the 98 studies, 75 were excluded for the following reasons: wrong publication type (i.e., systematic review and/or meta‐analysis), wrong population (i.e., primary diagnosis not related to PDs), wrong intervention (i.e., no psychotherapy provided), wrong outcome (i.e., absence of predictive factors for dropout), incorrect study design or non‐English publication.

**FIGURE 1:**
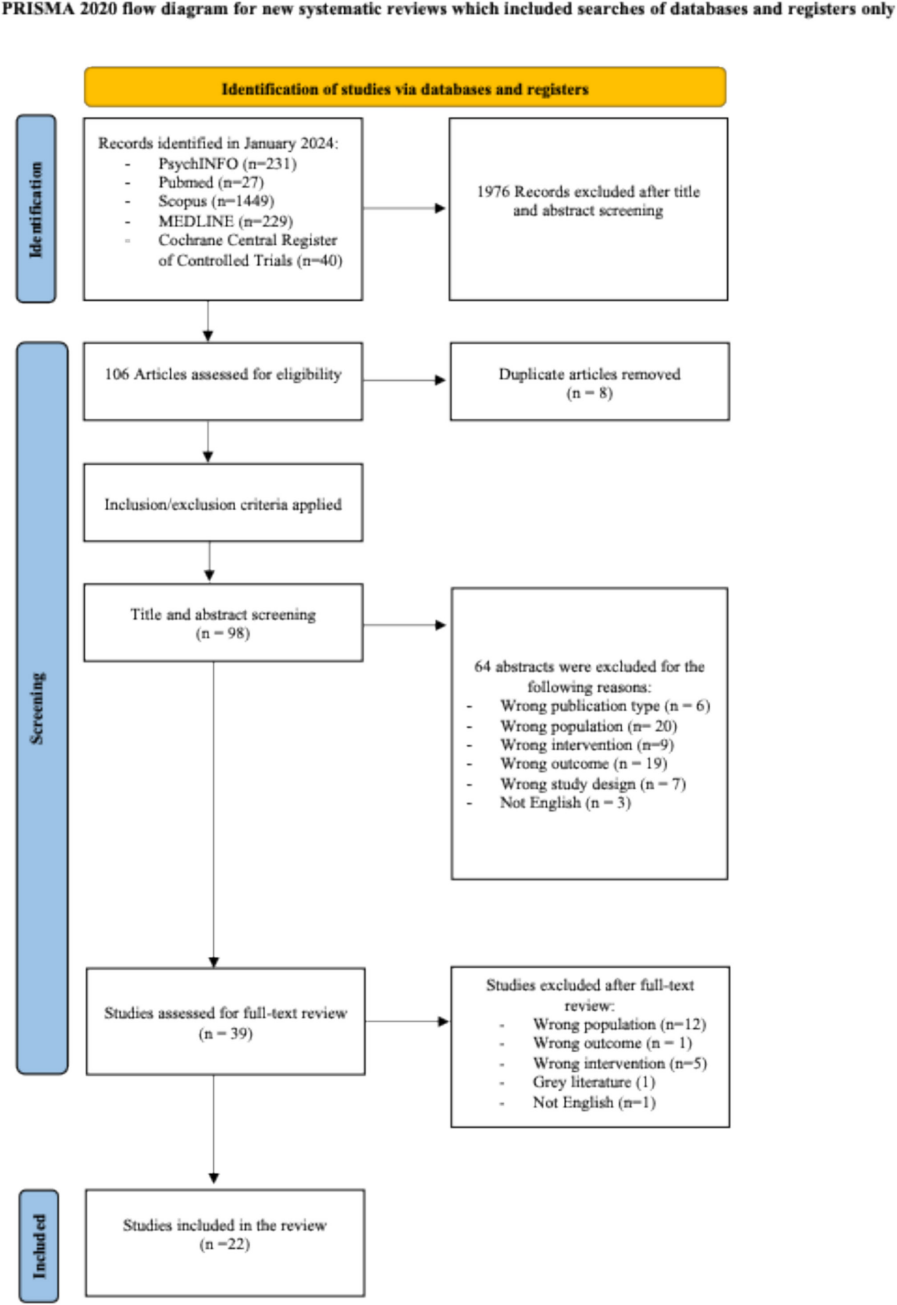
Prisma flow diagram.

Twenty‐two articles were finally included. All these articles were assessed for quality evaluation by applying the NOS. The process has been reported in Figure 1.

In the following paragraphs, study characteristics and results will be presented.

### 3.1 Studies Characteristics

Table 1 presents the characteristics of the studies based on the extraction parameters. The studies included in this review exhibit a sample size ranging from a minimum of 32 participants (Alesiani et al. 2014) to a maximum of 1423 participants (Kröger et al. 2014). Dropout rates ranged from 18% (Kröger et al. 2013) to 58% (Gaglia et al. 2013).

**TABLE 1:** Predictive variables of dropout in psychotherapy in individuals with personality disorders.

Authors (year)/nationality/NOS score^a^	Assigned sex	Mean age (SD)	Study design	Personality disorder diagnosis	Diagnostic approach	Construct measured	Type of treatment (average treatment duration)	Dropout rates	Primary outcomes	Secondary outcomes	Predictor for treatment discontinuation
Alesiani et al. (2014) Italy 7^a^	26 F 6 M	44.4 (9.3)	Longitudinal	NPD, HPD, PAPD and unspecified PD	DSM‐IV‐TR	Emotional regulation, personality traits, suicidal behaviours and hospitalizations	Systems Training for Emotional Predictability and Problem Solving (STEPPS) (2 years)	47%	Reduction in hospitalizations and suicidal attempts and improvement in emotional intensity regulation	Identification of personality traits associated with dropout	Histrionic personality trait, self‐transcendence and magical thinking
Arntz et al. (2015) Netherlands 8^a^	80 F 6 M	30.5 (7.8)	RCT	BPD	Structured Clinical Interview for DSM‐IV	Severity, dissociation, hostility and childhood physical abuse	ST and TFP (3 years, with twice‐weekly sessions)	27.3% (ST) 50.0% (TFP)	Recovery from BPD	Changes in BPD severity indices, dissociation burden and cost‐effectiveness	Hostility and childhood physical abuse
Barnicot et al. (2016) UK 7^a^	63 F 7 M	32 (10.6)	Longitudinal	BPD	Structured Clinical Interview for DSM‐IV	DBT skills use, therapeutic alliance, treatment credibility and self‐efficacy	DBT (1 year, twice‐weekly sessions)	46%	Frequency of self‐harm and dropout	Associations between DBT skills use, self‐efficacy and primary outcomes	None
Berghuis et al. (2021) Netherlands 8^a^	189 F 78 M	31.0 (9.14)	Naturalistic cohort study	Clusters A–C	DSM‐5	DSM‐5 personality traits (PID‐5) and dimensional personality disorder scores (PDQ‐4)	Psychodynamic and CBT oriented (NR)	18.4%	Dropout	Relationship between DSM‐5 personality traits and treatment engagement	Perceptual dysregulation, suspiciousness, rigid perfectionism, restricted affectivity, avoidant PD, psychological deterioration and commitment problems
Biała and Kiejna (2017) Poland 7^a^	80 F 30 M	NR	Prospective cohort study	Unspecified PD, BPD, NPD, HPD, PAPD, schizoid and dependent PDs	DSM‐IV criteria	Personality disorders, psychiatric diagnosis and treatment discontinuation	General inpatient psychiatric unit (pharmacotherapy, psychoeducation and art therapy), general psychiatric day‐care unit (diagnostic and rehabilitation focused) and neurosis treatment unit (psychodynamic group psychotherapy) (33.2, 31.5 and 90 days)	22.95%	Dropout	Relationship between specific personality disorder criteria and dropout risk	Identity diffusion, exaggerated and persistent complaints of misfortune (passive‐aggressive PD criterion) and borderline personality dimensions
Busmann et al. (2019) Germany 7^a^	56 F (completers) 39 M (completers) 26 F (noncompleters) 11 M (noncompleters)	29.7 (9.5) for completers, 28.5 (8.6) for noncompleters	Naturalistic longitudinal study	Clusters A–C	Structured Clinical Interview for DSM‐5 and LPFS for evaluating criterion A of AMPD	Personality functioning (DSM‐5 Criterion A), therapeutic alliance and symptom severity	Individual psychodynamic therapy, mentalization‐based group therapy, art therapy and DBT‐based skills training (80 days, 2 individual + 2 group sessions per week)	28%	Dropout	Personality functioning and therapeutic alliance	Impairments in self‐functioning (Criterion A of AMPD) and low therapist therapeutic alliance (positive collaboration, positive clinician impute and emotional difficulties)
Chalker et al. (2015) UK 7^a^	46 F 17 M	37.2 (10.4)	Longitudinal	BPD	Structured Clinician Interview for the DSM‐IV	Therapy‐interfering behaviours, self‐directed violence (SDV), psychological symptom severity and treatment satisfaction	DBT (NR)	57.1%	Dropout, frequency and severity of SDV	Emotion regulation deficits, client satisfaction, therapist satisfaction and changes in psychological symptoms	Frequency of out‐of‐session coaching contacts and avoidant/disengaged behaviours
Coutinho et al. (2014) Portugal 7^a^	26 F 12 M	29 (8.24)	Longitudinal	Cluster B and C	DSM‐IV	Therapeutic alliance and alliance rupture	CBT with principles of cognitive‐interpersonal therapy for personality disorders (NR)	44.7%	Patterns of therapeutic alliance and rupture markers over time	Differences between Axis I and Axis II disorders in alliance development and rupture events	Lower therapeutic alliance scores and raptures scores increased significantly over time and withdrawal scores
Doyle et al. (2023) Ireland 7^a^	90 F 12 M	33.65 (10.81)	Longitudinal	BPD	Structured Clinical Interview for DSM‐IV	Emotion dysregulation, impulsivity, BPD symptom severity, self‐harm, motivation and attachment style	DBT (NR)	34.3%	Dropout	Severity of BPD symptoms, self‐harm frequency and treatment readiness	Higher baseline scores on the Difficulties in Emotion Regulation Scale (DERS)
Euler et al. (2021) Germany 7^a^	309 F 24 M	31.96 (9.80)	Naturalistic longitudinal	BPD	Structured Clinical Interview for DSM‐IV	Childhood maltreatment (emotional/physical neglect and emotional/physical/sexual abuse), depressive symptoms, impulsivity and treatment retention	I‐DBT (NR)	25.8%	Treatment retention, changes in depressive symptoms and impulsivity	Association between specific childhood maltreatment types and treatment response	Emotional abuse and emotional neglect
Gaglia et al. (2013) UK 7^a^	90 F 12 M	32.4 (NR)	Retrospective cohort study	Clusters A–C	Structured Clinical Interview for DSM‐IV	Care coordination, substance misuse, personality disorder traits and treatment retention	DBT (1 year, weekly sessions + crisis coaching as needed)	58%	Dropout rates	Role of care coordination and clinical/demographic variables on dropout	Care coordination at the start of treatment and substance misuse
Hauber et al. (2020) Germany 7^a^	85 F 20 M	17.7 (1.7)	Longitudinal	Clusters A–C	DSM‐III	Therapeutic relationship (Child Session Rating Scale [C‐SRS]), dropout rates and session attendance	G‐MBT (6 months, 5 group + 2 individual sessions per week)	34.3%	Dropout rates and patterns of therapeutic relationship development	Role of therapeutic alliance in predicting dropout	Therapeutic Relationship declined significantly during the last three sessions for dropouts
Herzog et al. (2020) Germany 7^a^	731 F 165 M	31.1 (10.3)	Naturalistic longitudinal	BPD	DSM‐IV criteria	BPD symptom severity, functional outcomes (SF‐36) and treatment discontinuation	DBT (NR)	22.9%	Symptom‐specific change and functional improvement	Predictors of treatment discontinuation	Younger age, lower education, substance use disorder and recurrent depressive disorder
Jørgensen et al. (2021) Denmark 8^a^	89 F	15.98 (1.06)	RCT	BPD	DSM‐5 criteria	Borderline personality features, reflective functioning (RF), attachment to parents and peers, internalizing/externalizing symptoms and self‐harm	MBT‐G (1 year)	45%	Dropout	RF, attachment and clinical measures with dropout	Lower RF
Kröger et al. (2013) 7^a^	1075 F 348 M	32 (10.27)	Naturalistic longitudinal	BPD	Structured Clinical Interview for DSM‐IV	Borderline Symptom List, depressive symptoms, global functioning and dropout rates	DBT (3 months)	10.4%	Symptom reduction, improvement in depressive symptoms and psychosocial functioning	Response and remission rates and predictors of dropout	Younger age and substance use disorder
Kröger et al. (2014) Germany 7^a^	489 F 52 M	29 (8.24)	Naturalistic longitudinal	BPD	Structured Clinical Interview for DSM‐IV	Borderline Symptom List, depressive symptoms, dissociative symptoms and reasons for dropout or expulsion	DBT (3 months)	32.5%	Treatment retention and reasons for dropout and expulsion	Associations between clinical and sociodemographic factors with premature termination	Lack of motivation, repeated argument, inability to tolerate emotional distress and other critical life events
Remeeus et al. (2023) Germany 7^a^	94 F 20 M	30.8 (10.09)	Multicentre RCT	BPD	Structured Clinical Interview for DSM‐IV	Attachment dimensions (attachment anxiety and attachment avoidance), treatment engagement and dropout rates	MBT in two formats: day hospital (MBT‐DH) and intensive outpatient programme (MBT‐IOP) (18 months, respectively, 5 days per week and 2 days per week)	10.5%	Treatment dropout	Attachment dimensions	None
Steuwe et al. (2017) Germany 7^a^	68 F 21 M	29.8 (9.95)	Naturalistic longitudinal	BPD	DSM‐IV criteria	Borderline symptoms, depressive symptoms, PTSD, childhood trauma and therapeutic process variables	DBT (9.5 weeks)	24.7%	Treatment dropout	Association between comorbid PTSD, childhood trauma, therapist change and dropout	Therapist change, childhood emotional abuse, comorbid PTSD and physical neglect
Steuwe et al. (2023) Germany 7^a^	35 M 9 M	28.2 (8.60)	Naturalistic Longitudinal	BPD	DSM‐5 criteria	Borderline symptom severity, therapeutic alliance (WAI‐SR), childhood trauma and comorbidities	DBT (NR)	34.1%	Dropout	Therapeutic alliance, borderline symptom severity, depressive symptoms, general psychopathology, dissociative symptoms. quality of life and childhood trauma	Low therapeutic alliance and childhood trauma (emotional neglect and physical abuse)
Stratton et al. (2020) USA 7^a^	35 F 7 M	27.3 (7.45)	RCT	BPD	DSM‐IV criteria	Mindfulness, impulsivity, anger, depression, Axis I comorbidity and treatment retention	DBT (NR)	30.9%	Dropout	Mindfulness, impulsivity, anger and depression on dropout	Receiving disability benefits and mindfulness
Wnuk et al. (2013) Canada 7^a^	155 F 25 M	30.36 (9.9)	RCT	BPD	DSM‐IV criteria	Anger, impulsivity, therapeutic alliance, Axis I and Axis II comorbidity and lifetime suicide attempts	DBT and general psychiatric management (GPM) (1 year)	38%	Dropout	Anger, impulsivity, therapeutic alliance and clinical/demographic factors	Anger, lifetime suicide attempts and low therapeutic alliance

Abbreviations: AMPD = alternative model of personality disorder; BPD = borderline personality disorder; DBT = dialectical behavioural therapy; G‐MBT = group mentalization‐based therapy; HPD = histrionic personality disorder; I‐DBT = intensive dialectical behavioural therapy; LPFS = Level of Personality Functioning Scale; MBT = mentalization‐based therapy; NPD = narcissistic personality disorder; NR = not reported; PAPD = passive‐aggressive (negativistic) personality disorder; RCT = randomized controlled trial; ST = schema therapy; TFP = transference‐focused therapy.

^a^ Quality assessment was conducted using the Newcastle–Ottawa Scale (NOS), with a maximum score of 9 points for cohort and RCT designs (higher scores indicating higher quality). A modified version with a maximum of 8 points was used for cross‐sectional/longitudinal studies (Catalan et al. 2021).

Of the 22 studies extracted, 14 specifically focus on borderline PD (BPD) (Arntz et al. 2015; Barnicot et al. 2016; Busmann et al. 2019; Chalker et al. 2015; Doyle et al. 2023; Euler et al. 2021; Herzog et al. 2020; Jørgensen et al. 2021; Kröger et al. 2013, 2014; Remeeus et al. 2023; Steuwe et al. 2017; Steuwe et al. 2023; Stratton et al. 2020; Wnuk et al. 2013), while 8 focus on PDs more generally (Alesiani et al. 2014; Berghuis et al. 2021; Biała and Kiejna 2017; Busmann et al. 2019; Coutinho et al. 2014; Gaglia et al. 2013; Hauber et al. 2020; Herzog et al. 2020).

Regarding treatment approaches, 12 studies employed DBT (Barnicot et al. 2016; Chalker et al. 2015; Doyle et al. 2023; Euler et al. 2021; Gaglia et al. 2013; Herzog et al. 2020; Kröger et al. 2013; Steuwe et al. 2017, 2023; Stratton et al. 2020; Wnuk et al. 2013), 3 studies utilized dynamic psychotherapies (Biała and Kiejna 2017; Busmann et al. 2019; Wampers et al. 2018) and 3 studies applied MBT (Jørgensen et al. 2021; Remeeus et al. 2023). Additionally, one study conducted comparative analyses of treatments, specifically examining TFP versus schema therapy (Arntz et al. 2015), two studies used CBT (Alesiani et al. 2014; Coutinho et al. 2014) and the latest study used CBT or psychodynamic psychotherapy, based on the training of the therapists involved. The duration of treatment across these studies varied, with a minimum of 1 month (Euler et al. 2021) and a maximum of 18 months (Remeeus et al. 2023).

### 3.2 Predictive Variables of Dropout in BPD

Among the 22 studies reviewed, 14 investigated predictors of dropout in patients with BPD undergoing DBT (Arntz et al. 2015; Barnicot et al. 2016; Busmann et al. 2019; Chalker et al. 2015; Doyle et al. 2023; Euler et al. 2021; Herzog et al. 2020; Jørgensen et al. 2021; Kröger et al. 2013, 2014; Remeeus et al. 2023; Steuwe et al. 2017, 2023; Stratton et al. 2020; Wnuk et al. 2013). Two studies examined MBT (Busmann et al. 2019; Jørgensen et al. 2021), and one compared schema therapy with TFP. Dropout rates across these studies varied from 10.4% (Kröger et al. 2013) to 57.1% (Chalker et al. 2015).

In the DBT studies, key predictors of dropout included younger age (Herzog et al. 2020; Kröger et al. 2013), comorbid substance use disorders (Kröger et al. 2013), heightened emotional dysregulation (Doyle et al. 2023), difficulties in distress tolerance (Kröger et al. 2014) and elevated anger (Wnuk et al. 2013). Other factors, such as a history of childhood emotional abuse (Euler et al. 2021; Steuwe et al. 2017), therapist changes (Steuwe et al. 2017) and lack of motivation (Kröger et al. 2014), were also linked to increased dropout risk. Conversely, a strong therapeutic alliance (Wnuk et al. 2013; Steuwe et al. 2023), frequent use of DBT skills like mindfulness and emotion regulation (Barnicot et al. 2016) and engagement in phone coaching (Chalker et al. 2015) were associated with lower dropout rates. Socio‐economic factors, including receiving disability benefits (Stratton et al. 2020) and lower education levels (Herzog et al. 2020), were also predictors of higher dropout.

In MBT studies, the only significant predictor of dropout was lower reflective functioning (RF), with no other sociodemographic or clinical variables showing predictive value (Jørgensen et al. 2021). Remeeus et al. (2023) found that contrary to expectations, neither attachment avoidance nor attachment anxiety significantly impacted dropout rates. Finally, Arntz et al. (2015) compared schema therapy and TFP, revealing that hostility and childhood trauma were predictive of dropout in both treatments.

### 3.3 Predictive Variables of Dropout in PDs

Among the 22 studies reviewed, eight focused on a general population of patients with PDs in general (Alesiani et al. 2014; Berghuis et al. 2021; Biała and Kiejna 2017; Busmann et al. 2019; Coutinho et al. 2014; Gaglia et al. 2013; Hauber et al. 2020; Herzog et al. 2020). Dropout rates between these studies varied from 22.9% (Alesiani et al. 2014) and 47% (Herzog et al. 2020).

Busmann et al. (2019) investigated predictors of treatment dropout in inpatients undergoing a combination of short‐term psychodynamic psychotherapy and mentalization‐based group therapy. They identified that greater impairments in self‐functioning and a weaker therapeutic alliance, as rated by the therapist, significantly increased the likelihood of premature treatment discontinuation. Berghuis et al. (2021) focused on pretreatment predictors of dropout in a (day) clinical group psychotherapy programme using either a psychodynamic or CBT approach. They found that high scores on maladaptive personality traits especially in patients with Clusters B and C PDs (e.g., perceptual dysregulation, unusual belief and experiences, suspiciousness, rigid perfectionism and restricted affectivity) significantly predicted dropout. Wampers et al. (2018) highlighted the role of lower educational attainment, Cluster A PDs and the total number of Axis I and Axis II diagnoses as factors associated with dropout in a psychodynamic hospitalization‐based programme, with the number of Axis II diagnoses being the only statistically significant predictor.

Alesiani et al. (2014) examined dropout rates in an inpatient cohort undergoing Systems Training for Emotional Predictability and Problem Solving (STEPPS) for BPD. They reported a 47% dropout rate and found that histrionic personality traits and magical thinking were significant predictors of dropout, suggesting that specific personality characteristics may interfere with treatment retention. Biała and Kiejna (2017) assessed the influence of PDs on dropout rates from psychiatric hospitalization across three units, finding that although 22.95% of patients dropped out, the presence of PDs alone did not significantly predict dropout. However, the organization of treatment in different units seemed to influence this outcome, particularly among patients with borderline and passive‐aggressive PDs. The authors highlighted that psychodynamic group psychotherapy can be the most adequate treatment for patients with personality problems. Coutinho et al. (2014) explored the role of therapeutic alliance in dropout during CBT for patients with both Axis I and Axis II disorders. The study showed that patients with PDs began with lower alliance scores that worsened over time, which correlated with higher dropout rates. In contrast, patients with Axis I disorders showed improved alliance scores and better retention, emphasizing the importance of a strong therapeutic relationship. Gaglia et al. (2013) studied dropout rates in DBT within the UK's National Health Service, reporting a high dropout rate of 58%. Care coordination was significantly associated with higher dropout rates, with 88% of patients receiving coordinated care dropping out compared to 52% without such care, suggesting that systemic factors like care coordination might reduce engagement in DBT.

Finally, Hauber et al. (2020) examined the relationship between the therapeutic alliance and dropout in an intensive mentalization‐based group therapy programme for adolescents with PDs. Their findings indicated that while both dropouts and completers started with similar alliance scores, dropouts experienced a significant decrease in these scores by the end of treatment. This highlights the importance of continuously monitoring the therapeutic relationship to prevent premature termination.

## 4 Discussion

This systematic review offers a comprehensive overview of predictive factors associated with dropout from psychotherapy in patients with PDs, with a particular emphasis on BPD. Key predictors of dropout for patients with BPD include younger age, comorbid substance use disorders, emotional dysregulation and difficulties with distress tolerance (Herzog et al. 2020; Kröger et al. 2013, 2014; Doyle et al. 2023; Wnuk et al. 2013). Additionally, factors such as a history of childhood emotional abuse, therapist changes and lack of motivation also contribute to an increased dropout risk (Euler et al. 2021; Steuwe et al. 2017; Kröger et al. 2014).

Conversely, a strong therapeutic alliance, the use of skills like mindfulness and engagement in phone coaching are associated with lower dropout rates (Wnuk et al. 2013; Steuwe et al. 2023; Barnicot et al. 2016; Chalker et al. 2015). Other relevant factors include low RF and socio‐economic challenges, such as receiving disability benefits and lower education levels (Jørgensen et al. 2021; Stratton et al. 2020; Arntz et al. 2015).

Dropout rates in psychotherapy for PDs remain a significant challenge, adversely affecting both treatment outcomes and the broader mental health system. The meta‐analytic study by Iliakis et al. (2021) highlighted a dropout rate of approximately 22.3% across all BPD psychotherapy studies, which increased to 28.2% in outpatient randomized controlled trials (RCTs). These dropout rates suggest a consistent pattern of disengagement from therapy that adversely affects both patient outcomes and resource allocation within healthcare systems. Moreover, a multilevel survival meta‐analysis conducted by Arntz et al. (2023) delves deeper into the dynamics of dropout, showing that group therapy formats and community treatment by experts are associated with higher dropout rates compared to individual therapy. This trend aligns with findings from Cooper et al. (2023), who emphasized that dropout occurs early in treatment and is not confined to specific treatment modalities, impacting established interventions such as DBT and MBT. Efforts to improve retention should, therefore, focus on the critical early stages of treatment.

The reasons for dropout, as noted across the studies, often include patient dissatisfaction, lack of motivation or expulsion from treatment due to failure to adhere to therapy protocols. Iliakis et al. (2021) pointed out that dropout is particularly prevalent in outpatient settings, possibly due to the less structured nature of these environments compared to inpatient or partial hospitalization settings. Additionally, factors such as poor therapeutic alliance and comorbidities, especially substance use, have been identified as major contributors to dropout.

In summary, despite advances in evidence‐based therapies for PDs, such as MBT or DBT, dropout rates remain problematic (Bornovalova and Daughters 2007; McMurran et al. 2010). For this reason, paying attention to protective factors is imperative. From this systematic review therapeutic alliance (Busmann et al. 2019; Coutinho et al. 2014; Hauber et al. 2020; Steuwe et al. 2017, 2023) and RF (Jørgensen et al. 2021) emerged as variables strongly implicated in inverting dropout phenomena. RF, which reflects the capacity to understand and interpret one's own and others' mental states, is often impaired in PDs and other clinical conditions (Ball Cooper et al. 2021; Bateman and Fonagy 2004; De Salve et al. 2023; Fonagy and Bateman 2016), leading to challenges in emotional regulation, interpersonal trust and treatment adherence. Enhancing RF fosters self‐awareness, emotional resilience and collaborative engagement, all of which are critical for maintaining motivation in therapy (Bateman and Fonagy 2013; Fonagy and Bateman 2016). However, empirical evidence linking RF to dropout remains limited. Only one study included in the review (Jørgensen et al. 2021) has specifically examined and found a significant effect of RF on dropout in MBT. Other MBT studies have not explored the predictive role of RF but have instead focused on different dropout‐related factors, such as the therapeutic alliance (Hauber et al. 2020) and attachment dimensions (Remeeus et al. 2023). This difference in research focus suggests that the lack of findings on RF in other MBT studies does not necessarily imply that RF is unrelated to dropout but rather that it has not been extensively investigated within this framework. Several factors may influence the relationship between RF and dropout in psychotherapy. Variability in patient characteristics (e.g., personality pathology severity, attachment styles and baseline RF) may moderate the impact of RF on treatment retention (Reatto et al. 2023). Additionally, differences in study designs and assessment methods could account for inconsistencies in findings. Therapist‐related factors, such as the clinician's own mentalizing ability and their approach to fostering RF in patients (Rogoff et al. 2021), might also play a crucial role in shaping treatment retention.

To reduce the risk of dropout, interventions should emphasize early engagement and individualized treatment approaches for high‐risk patients. Establishing a strong therapeutic alliance from the beginning is crucial, along with utilizing strategies such as motivational interviewing to foster commitment to therapy. Pretreatment preparation, including structured orientation sessions, can help set realistic expectations and support patients in adapting to the therapeutic process. Additionally, integrating digital tools, such as mobile applications for skills practice and scheduled remote check‐ins with therapists, may enhance engagement, particularly for individuals struggling with emotional dysregulation or difficulty managing distress. For patients with co‐occurring substance use disorders, incorporating harm‐reduction techniques and strengthening collaboration with addiction specialists may help sustain participation in treatment. Offering greater flexibility in session scheduling and delivery formats, such as a combination of in‐person and remote sessions, could also address socio‐economic challenges that contribute to therapy discontinuation. These targeted strategies, with a focus on RF and therapeutic alliance, are essential for improving treatment adherence and outcomes in this population.

Despite these challenges, RF and the therapeutic alliance remain interrelated constructs, both of which are central to treatment engagement. The therapeutic alliance—a collaborative and trusting relationship between therapist and patient—is a well‐established predictor of treatment retention and outcomes (Lingiardi et al. 2011; Tanzilli and Gualco 2020). For patients with PDs, who frequently struggle with relational instability and mistrust, a strong alliance mitigates relational ruptures and provides a secure base for exploration and change. Importantly, RF and therapeutic alliance mutually reinforce each other, with improved RF strengthening the alliance and a robust alliance creating a safe space for RF to develop. Together, these factors act as buffers against common dropout triggers, such as emotional overwhelm or therapeutic impasses, underscoring their critical role in sustaining engagement and enhancing psychotherapy outcomes. Addressing dropout requires a multifaceted approach, including improving early engagement, offering flexible therapy formats and tailoring interventions to individual patient needs.

### 4.1 Limitations and Future Directions

This systematic review of predictors of dropout from psychotherapy among patients with PDs has several limitations that warrant consideration. First, the included studies exhibit considerable heterogeneity in their designs, sample populations and outcome measures, which complicates the synthesis of results and their generalizability across diverse patient groups. This heterogeneity limits the ability to directly compare results across studies and affects the generalizability of findings to broader clinical populations. Future research should aim for greater methodological consistency to enhance comparability and strengthen the robustness of conclusions.

Second, there is an overrepresentation of BPD in the reviewed studies, which may limit the applicability of findings to other PDs. While dropout predictors have been well explored in BPD populations, there is a lack of studies examining dropout in individuals with Cluster A (e.g., schizotypal and paranoid) and Cluster C (e.g., avoidant and obsessive–compulsive) PDs. These conditions may have distinct predictors of dropout, influenced by factors such as social withdrawal, anxiety or rigidity in interpersonal relationships. Future studies should aim to address this gap by examining dropout predictors across a broader range of PDs to provide a more comprehensive understanding of psychotherapy disengagement.

Furthermore, the potential for publication bias exists, as studies with significant results are more likely to be published than those with null findings, skewing the overall conclusions. The quality of evidence also varies, with many studies potentially subject to methodological flaws and biases. Additionally, many studies may employ short follow‐up periods, failing to capture long‐term dropout trends and mechanisms. The review may also lack a thorough exploration of the underlying mechanisms by which identified predictors influence dropout. For this reason, more longitudinal studies focusing on therapist–patient dyads, incorporating patient and therapist characteristics, should be implemented in the near future (De Salve et al. 2024). Few studies have focused on therapist characteristics, even though results show that therapist interpersonal functioning and skills have the strongest evidence of a direct effect on treatment outcomes (Lingiardi et al. 2018).

Lastly, while findings from controlled studies offer insights, their applicability to real‐world clinical settings may differ, emphasizing the need for further research that explores dropout across various populations and contexts, as well as the patient experiences influencing treatment engagement.

Nevertheless, it is believed that this systematic review has the potential to highlight important aspects capable of informing clinical practice. By synthesizing evidence from various studies, it may provide valuable insights that can guide therapeutic interventions and decision‐making processes, ultimately enhancing the quality of care for patients.

## 5 Conclusion

The findings from the systematic review highlight the importance of adjusting therapeutic interventions according to individual patient characteristics to improve outcomes and reduce the risk of premature therapy dropout. Andrews and Dowden (2007) distinguish between two types of therapeutic responsiveness: the overall effectiveness of the intervention and the adaptation of treatment to the specific needs of the patient, including sociodemographic, clinical and motivational factors. The authors emphasize the value of pretreatment assessments to identify patients at risk of dropout, focusing not only on sociodemographic variables but also on factors such as clinical history and engagement in care coordination programmes.

de Jong et al. (2021) underscore the importance of sustained therapeutic collaboration, where the therapist is actively involved in monitoring the quality of the therapeutic relationship. Complementing this, McMain et al. (2015) suggest five key strategies for reinforcing the therapeutic alliance, which include promoting emotional awareness and implementing a structured approach to therapy. These strategies are designed to foster a secure and supportive environment, allowing both therapist and client to navigate challenges collaboratively and enhance the therapeutic process. The therapist's ability to recognize and manage alliance ruptures is especially crucial when working with borderline patients, who are known for emotional volatility, a significant risk factor for dropout (McMain et al. 2015). While this characteristic is often associated with borderline disorders, establishing a robust therapeutic alliance is essential for patients with all PD diagnoses, as adapting therapy can improve engagement and reduce the risk of therapeutic failure (de Freixo Ferreira et al. 2023; McMain et al. 2015).

In conclusion, this systematic review provides a thorough overview of the literature, synthesizing findings from various studies to illuminate the factors influencing dropout rates in PDs, particularly among individuals with BPD. It underscores the pressing need for responsive interventions in treating PDs to mitigate the high rates of premature therapy dropout. Recognizing predictors such as therapeutic alliance strength, patient age, emotional regulation capabilities and comorbidities like substance use presents an opportunity to enhance engagement, particularly during the critical early stages of treatment.

These findings have important implications for mental health policies aimed at improving patient retention in psychotherapy. Healthcare systems should prioritize resources for early identification of patients at higher risk of dropout and support targeted interventions to address their specific needs. This may include investing in pretreatment assessment tools, developing structured therapist training programmes on alliance building and dropout prevention and incorporating these strategies into official clinical guidelines. Ensuring accessibility through flexible treatment formats, financial assistance for vulnerable populations and better coordination between mental health services may also play a key role in reducing premature discontinuation of therapy.

Furthermore, the integration of recent research ensures that the conclusions drawn are based on current evidence, enhancing the validity and relevance of the findings. The emphasis on clinical implications underscores the necessity for customized psychological interventions to improve efficacy and reduce early dropout. Concerning future research, particularly the need for longitudinal studies focusing on therapist–patient dyads, points to important gaps in the literature.

While dropout remains a complex, multifaceted issue, the evidence points to specific, actionable strategies—such as strengthening emotional awareness and maintaining structured therapeutic support (McMain et al. 2015; de Jong et al. 2021)—that can foster resilience within the therapeutic relationship. Addressing both clinical and systemic factors related to dropout could lead to more effective engagement strategies, ultimately improving treatment retention and outcomes for individuals with PDs. Continued exploration into the dynamics of therapist–patient dyads through longitudinal research will be essential for refining these approaches and, ultimately, improving treatment adherence and outcomes in this challenging patient population.

## Author Contributions

The conceptualization of the study was carried out by F.D.S., who developed the research framework and objectives. F.D.S. and E.G. designed the study, screened and selected the studies, read the full‐text articles and extracted data from the included studies. C.R. ensured the studies' scientific rigour through the risk of bias assessment and reviewed and edited the manuscript. Project administration and supervision were overseen by O.O. and I.M.

## Conflicts of Interest

The authors declare no conflicts of interest.

## Data Availability

Data sharing is not applicable to this article as no datasets were generated or analysed during the current study.
